# Book Review: Drug Hypersensitivity by W. J. Pichler (Bern)

**DOI:** 10.1097/WOX.0b013e3181655dde

**Published:** 2008-02-15

**Authors:** Johannes Ring

**Affiliations:** 1 Munich, Germany

## 

438 pages, 78 figures, Hard Cover, ISBN 978-3-8055-8269-8, Karger, Basel (2007)

Drug hypersensitivities represent an increasing problem in clinical medicine. They manifest under colorful and variable symptoms as exanthematous eruptions, fever, and affection of internal organs, and also as anaphylactic reactions. Drug hypersensitivities also are a challenging field of research because they induce a broad spectrum of clinical symptoms through quite different pathophysiological mechanisms; part of them are not very well understood.

The volume by Werner Pichler represents a phenomenal approach to cover the whole field from epidemiology, clinical symptoms, pathophysiology, to diagnostic and therapeutic workup. The author manages not only to include immunoglobulin E-dependent reactions, but also many other types of pathogenic immune reactions. In the section on diagnostic methods, new approaches with cellular in vitro techniques are discussed. In 33 chapters, with outstanding experts as authors, the book covers several aspects: starting with epidemiology and prevalence of the different types of adverse drug reactions. In the chapter "Pathomechanisms," genetics and animal models, molecular characteristics of drugs as haptens, special problems related to drugs, human immunodeficiency virus, and acquired immune deficiency syndrome are discussed. In the chapter "Clinical Manifestations," the problems of urticaria and anaphylaxis, analgesics, radiographic contrast media, and antibiotics are covered. Organ-related damages such as drug allergic liver injury, nephritis, or blood dyscrasias are equally considered.

In the outlook, strategies of desensitization are discussed. More and more often, the situation arises where patients are allergic to a life-saving drug that cannot be stopped. This occurs especially in severe infections, but also in oncology.

This book is of immense value not only to allergists and dermatologists, but also to all physicians prescribing medication, to scientists, and to the pharmaceutical industry.

Johannes Ring, MD, PhD

Munich, Germany

